# Longitudinal functional brain network reconfiguration in healthy aging

**DOI:** 10.1002/hbm.25161

**Published:** 2020-08-28

**Authors:** Brigitta Malagurski, Franziskus Liem, Jessica Oschwald, Susan Mérillat, Lutz Jäncke

**Affiliations:** ^1^ University Research Priority Program “Dynamics of Healthy Aging” University of Zurich Zurich Switzerland; ^2^ Division of Neuropsychology, Institute of Psychology University of Zurich Zurich Switzerland

**Keywords:** brain networks, healthy aging, multilayer modularity, network flexibility, resting‐state fMRI

## Abstract

Healthy aging is associated with changes in cognitive performance and functional brain organization. In fact, cross‐sectional studies imply lower modularity and significant heterogeneity in modular architecture across older subjects. Here, we used a longitudinal dataset consisting of four occasions of resting‐state‐fMRI and cognitive testing (spanning 4 years) in 150 healthy older adults. We applied a graph‐theoretic analysis to investigate the time‐evolving modular structure of the whole‐brain network, by maximizing the multilayer modularity across four time points. Global flexibility, which reflects the tendency of brain nodes to switch between modules across time, was significantly higher in healthy elderly than in a temporal null model. Further, global flexibility, as well as network‐specific flexibility of the default mode, frontoparietal control, and somatomotor networks, were significantly associated with age at baseline. These results indicate that older age is related to higher variability in modular organization. The temporal metrics were not associated with simultaneous changes in processing speed or learning performance in the context of memory encoding. Finally, this approach provides global indices for longitudinal change across a given time span and it may contribute to uncovering patterns of modular variability in healthy and clinical aging populations.

## Graphical abstract

In this research paper, we used a longitudinal dataset consisting of four occasions of resting‐state‐fMRI and cognitive testing (spanning 4 years) in 150 healthy older adults. We applied a graph‐theoretic analysis to investigate the time‐evolving modular structure of the whole‐brain network, by maximizing the multilayer modularity across four time points. Network flexibility, which reflects the tendency of brain nodes to switch between modules across time, was significantly higher in older adults than in a null model and with increasing age, indicating that older age is related to increased variability in modular organization.

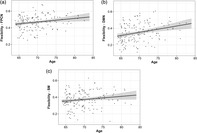

## 1 INTRODUCTION

It is well‐known that during resting state, the human brain is composed of several distinct and distributed functional subnetworks (i.e., modules) of densely interconnected brain regions sparsely connected to the rest of the network (Wig, 2017). Previous studies have consistently identified several major modules, such as the default mode (DMN), executive control, salience, sensorimotor, and visual (VIS) networks (Heine et al., 2012; Smitha et al., 2017; van den Heuvel & Hulshoff Pol, 2010). This modular organization affords the brain a superior resilience to disease or injury as the individual modules can adapt to the changing environment or pathology without compromising the rest of the subnetworks (Fornito, Zalesky, & Breakspear, 2015; Stam, 2014).

Importantly, the architecture of these subnetworks has been shown to undergo important developmental and aging‐related changes (Baum et al., 2017; Fair et al., 2009; Gu et al., 2015), with previous studies suggesting reduced functional segregation with advancing age (Cao et al., 2014; Chan, Park, Savalia, Petersen, & Wig, 2014; Geerligs, Renken, Saliasi, Maurits, & Lorist, 2015; Malagurski, Liem, Oschwald, Mérillat, & Jäncke, 2020; Müller, Mérillat, & Jäncke, 2016; Song et al., 2014), and increased modular variability or heterogeneity within higher order cortices in healthy elderly (Iordan et al., 2018; Peraza, O'Brien, Blamire, Kaiser, & Taylor, 2018; Schlesinger, Turner, Lopez, Miller, & Carlson, 2017).

Based on such studies, we can conclude that functional connectivity within higher order resting state networks (i.e., DMN) decreases while, between‐network connectivity (i.e., DMN and executive control network) increases, resulting in less modular, “dedifferentiated” brains in older age (Chan et al., 2014; Ng, Lo, Lim, Chee, & Zhou, 2016).

In addition, these changes have been associated with less efficient cognitive functioning, highlighting the critical importance of this organizational characteristic of the human brain (Iordan et al., 2018; Gallen et al., 2017; Geerligs et al., 2015; Chan et al., 2014; Müller et al., 2016).

Although these studies have provided important insights into modular reorganization, most of them are, however, based on cross‐sectional data and, thus, fail to describe true longitudinal change in advanced aging.

Recent work in network neuroscience has given rise to multilayer network modeling suitable to track the evolution of modular reconfiguration throughout time (Braun et al., 2018; de Domenico, 2017). This framework is more powerful in contrast to more traditional approaches as it can provide an aggregate measure of change across multiple time points, such as network flexibility, which characterizes how frequently brain regions switch allegiance from one module to another over time (Bassett et al., 2011). Although still in its infancy, this approach has been used to investigate the dynamic (within‐session) modular structure during resting state (Betzel, Satterthwaite, Gold, & Bassett, 2017; Gerraty et al., 2018; Shine, Koyejo, & Poldrack, 2016), task performance (Bassett et al., 2011; Bassett, Yang, Wymbs, & Grafton, 2015; Braun et al., 2015; Schlesinger et al., 2017; Telesford et al., 2017), development (Betzel et al., 2015), and brain disorders (Braun et al., 2016), showing interesting patterns of flexible network reconfiguration dependent on the “task at hand.” Indeed, one study has used multilayer networks to investigate how the brain's modular organization evolves across the human lifespan, suggesting that some modules tend to be highly flexible and exhibit substantial reconfiguration throughout adulthood (Betzel et al., 2015).

For the present study, we used a longitudinal dataset comprised of four occasions of resting state functional brain imaging and cognitive testing in healthy older adults. We applied the multilayer modularity model to investigate how the brain's modular structure changes in healthy aging over a span of 4 years. Further, based on the previous studies connecting this functional property to cognitive functioning, we assessed how modular reconfiguration relates to changes in cognitive performance. More specifically, we calculated the functional flexibility, promiscuity, cohesion strength, and disjointedness to study the degree to which brain regions switch between communities as well as the pattern of it, and the recruitment coefficient to study the association of the obtained community structure to well‐known resting state networks.

Moreover, we explored the relationship between functional flexibility and (a) processing speed and (b) learning performance in the context of memory encoding. These two cognitive domains were chosen as they have been found to be particularly vulnerable to aging effects (Salthouse, 2010; Schaie, 2005) and have been previously related to brain modularity in healthy elderly (Gallen et al., 2017; Geerligs et al., 2015; Iordan et al., 2018; Ng et al., 2016).

We hypothesized that the functional networks would show high flexibility across the time span of 4 years and with increasing age, suggesting longitudinal modular reconfiguration and instable modular architecture in older adults. Finally, we assumed that greater flexibility is associated with poorer performance in both cognitive domains.

## 2 METHODS

### 2.1 Participants

Longitudinal resting‐state fMRI (rs‐fMRI) data were taken from the Longitudinal Healthy Aging Brain Database Project (LHAB; Switzerland)—an ongoing project conducted at the University of Zurich (Zöllig et al., 2011). We used data from the first four measurement occasions (baseline, 1‐year follow‐up, 2‐year follow‐up, 4‐year follow‐up). The baseline dataset included 232 participants (age at baseline: *M* = 70.8, range = 64–87; females: 114). At each measurement occasion, participants completed an extensive battery of neuropsychological and psychometric cognitive tests and underwent brain imaging. The brain imaging session was conducted in close temporal proximity to the behavioral assessments (difference between behavioral and MRI assessments in days [*M* ± *SD*]: baseline: 2.2 ± 5.2, 1‐year follow‐up: 2.6 ± 5.2, 2‐year follow‐up: 4.3 ± 13.0, 4‐year follow‐up: 4.6 ± 9.3). Inclusion criteria for study participation at baseline were age ≥64, right‐handedness, fluent German language proficiency, a score of ≥26 on the Mini Mental State Examination (Folstein, Folstein, & McHugh, 1975), no self‐reported neurological disease of the central nervous system and no contraindications to MRI. The study was approved by theethical committee of the canton of Zurich. Participation was voluntary and all participants gave written informed consent in accordance with the declaration of Helsinki.

For the present analysis, we only included participants with complete rs‐fMRI data (four measurement occasions). This was necessary because four temporal windows enable a more reliable maximization of the multilayer modularity function. At 4‐year follow‐up, the dataset still comprised 74.6% of the baseline sample (*n* = 173), of which 86.7% (*n* = 150, age at baseline: *M* = 69.8, range = 64–83; females: 71) had complete data for rs‐fMRI.

To estimate whether attrition was selective, we compared the full sample at baseline with participants that had rs‐fMRI data from all four measurement occasions. The total selectivity was computed by standardizing the difference between the mean in the baseline sample and the sample with no missing data, on the *SD* of the baseline sample in the variable of interest (Lindenberger, Singer, & Baltes, 2002).

The size of the selectivity index was interpreted with reference to an effect size. As it can be seen in Supplementary Table S1, the total selectivity was negligible for all measures (i.e., none of the measures exceeded the cutoff of 0.20 for a weak effect according to Cohen (1988)), indicating that the participants with no missing rs‐fMRI data did not significantly differ from the full baseline sample in terms of age at baseline, education, initial cognitive ability, or physical and mental health.

### 2.2 Neuropsychological assessment

All participants completed a neuropsychological test battery assessing multiple cognitive domains at each measurement occasion. For the present analysis, data from the domains “processing speed” and “learning/memory encoding” were used. Individual scores were standardized to T scores (*M* = 50, *SD* = 10) with respect to baseline and averaged across subtests to calculate the domain‐average composite scores.

*Processing speed* was assessed using four psychometric paper‐pencil tests: (a) the number of correct responses across two test parts of the Identical Pictures Test (Kit of Factor‐Referenced Cognitive Tests; Ekstrom, French, Harman, & Dermen, 1976); (b) the number of correct responses (within 2 min) on the Digit Symbol Test (Wechsler Intelligence Scale for Adults; Von Aster, Neubauer, & Horn, 2006); (c) time in seconds, including the time, used when an error was made, needed to finish the Trail‐Making‐Test A (Reitan & Wolfson, 2004) (the scores were reversed so that the higher scores equaled better performance); and (d) number of correct responses (within 2 min) on the LPS14, a subtest from the Leistungsprüfsystem 50+ (LPS), a German intelligence test developed to measure Thurstone's (1938) primary mental abilities (Horn, 1983).

*Learning/Memory encoding* was defined using the (a) number of correctly reproduced abstract designs at first five trials of the DCS figural memory test (Diagnosticum für Cerebralschädigung; Weidlich & Lamberti, 2001), and (b) total correct responses over five immediate free recall trials from the Verbal Learning and Memory Test (Helmstaedter & Durwen, 1990), a German equivalent of the Rey Auditory Verbal Learning Test.

### 2.3 MRI acquisition

MRI scans were acquired at the University Hospital of Zurich on a Philips Ingenia 3T scanner (Philips Medical Systems, Best, The Netherlands). T1‐weighted (T1w) structural images were acquired using a gradient echo sequence (3D turbo field echo, 160 slices, TR = 8.1 ms, TE = 3.7 ms, FOV = 240 × 240 × 160 mm, flip angle = 8°, isotropic voxel size = 1.0 × 1.0 × 1.0 mm^3^). Two hundred and twenty‐five multislice T2*‐weighted volumes were retrieved with a gradient echo‐planar sequence using transverse slice orientation (43 slices; voxel size: 3.5 × 3.5 × 3.5 mm^3^; TR = 2,000 ms; TE = 21 ms; flip angle = 76°; FOV = 220 × 220 × 150 mm).

### 2.4 MRI preprocessing

Preprocessing was performed using the fmriprep BIDS app (v.1.0.5) (Esteban et al., 2019; Gorgolewski et al., 2017), a Nipype (Gorgolewski et al., 2011) based tool. Each T1w (T1‐weighted) volume was corrected for INU (intensity nonuniformity) using N4 Bias Field Correction v.2.1.0 (Tustison et al., 2010) and skull‐stripped using ANTs v.2.1.0 (using the OASIS template). Spatial normalization to the ICBM 152 Nonlinear Asymmetrical template version 2009c (Fonov, Almli, Evans, Collins, & McKinstry, 2009) was performed through nonlinear registration with the antsRegistration tool of ANTs v.2.1.0 (Avants, Epstein, Grossman, & Gee, 2008), using brain‐extracted versions of both T1w volume and template. Brain tissue segmentation of cerebrospinal fluid (CSF), white matter (WM), and gray matter (GM) was performed on the brain‐extracted T1w using FAST (Zhang, Brady, & Smith, 2001) (FSL v5.0.9). Functional data was slice time corrected using 3dTshift from AFNI v.16.2.07 (Cox, 1996) and motion corrected using mcflirt (FSL v5.0.9; Jenkinson, Bannister, Brady, & Smith, 2002). This was followed by co‐registration to the corresponding T1w using boundary‐based registration (Greve & Fischl, 2009) with 9° of freedom, using flirt (FSL). Motion correcting transformations, BOLD‐to‐T1w transformation, and T1w‐to‐template (MNI) warp were concatenated and applied in a single step using antsApplyTransforms (ANTs v.2.1.0) using Lanczos interpolation.

Further preprocessing steps were performed using the CONN toolbox (Whitfield‐Gabrieli & Nieto‐Castanon, 2012). The nuisance regressors were defined according to the 36‐parameter model (Ciric et al., 2017): six motion parameters, signals estimated from CSF and WM, global signal, their derivatives, quadratic terms, and squares of derivatives were regressed out from functional data separately for each run. The rs‐fMRI data was temporally bandpass filtered in the 0.01–0.1 Hz frequency range. We applied simultaneous filtering/nuisance regression, because it was shown to reduce correlation between time‐series fluctuations and motion (Hallquist, Hwang, & Luna, 2013). Global signal regression was performed in accordance with previous studies on aging (Chan et al., 2014; Ng et al., 2016), as this has been shown to be effective in the reduction of the effects of physiological signals and head motion (Lydon‐Staley, Ciric, Satterthwaite, & Bassett, 2019).

### 2.5 Network definition

The regions of interest (ROIs), used to build the network, were selected from the atlas of Schaefer and colleagues (Schaefer et al., 2018), corresponding to 200 cortical regions (i.e., ROIs) classified into seven well‐known resting state networks according to the Yeo‐Krienen atlas (Yeo et al., 2011): frontoparietal control (FPCN), default mode (DMN), dorsal attention (DAN), salience ventral attention (SVAN), limbic (LIMB), somatomotor (SM), and visual (VIS) networks.

The time‐series were averaged over the voxels in each ROI and correlated between each pair of nodes using the Pearson's correlation analysis. Each correlation coefficient was then Fisher's *r*‐to‐*z* transformed. Due to ambiguity regarding the meaning of negative correlations in the context of global signal regression (Murphy & Fox, 2017), negative z values were excluded from the analysis in accordance to previous studies (Chan et al., 2014, 2018). This resulted in subject‐specific 200 × 200 correlation matrices with diagonal and negative values set to zero. Therefore, the main network analysis was performed on positive weighted networks.

In addition, supplementary analysis has been done on positive weighted networks containing only statistically significant connections at an FDR‐adjusted significance level of *p* < .01. This has been done to ensure that our results are robust in both unthresholded and thresholded matrices based on the significance level of node connections.

### 2.6 Multilayer modularity

Modular structure in a network indicates that nodes in a module are more interconnected with one another then with the rest of the network. Modules within complex networks are identified using community detection algorithms such as the maximization of the modularity quality function (i.e., Q; Newman & Girvan, 2004). These algorithms are applicable to traditional single layer networks (e.g., within‐session static functional connectome) or multilayer networks in which nodes are connected across layers (Kivelä et al., 2014). These layers may correspond to different modalities (e.g., structural and functional connections between brain regions) or different temporal instants at which the network was observed (i.e., brain connectivity across a span of several years). So, in addition to being able to calculate single window modularity scores, the multilayer framework is interesting for longitudinal studies because it can produce a “global index” which reflects the changes in network organization across a defined period (i.e., 4 years). In the present study, we define multilayer networks where each layer is the functional connectivity matrix at a given time point, resulting in a four‐layer temporal network for each of the subjects, separately.

Thus, to investigate the changes in network organization across time, the multilayer modularity was optimized (Blondel, Guillaume, Lambiotte, & Lefebvre, 2008; Mucha, Richardson, Macon, Porter, & Onnela, 2010) as follows:

$$Q=\frac{1}{2\mu }\;\sum_{\text{ijlr}}\left\{\;\left(A_{\mathrm{ijl}}-\gamma_{l}P_{\mathrm{ijl}}\right)\;\delta_{\mathrm{lr}}+\delta_{\mathrm{ij}}\omega_{\mathrm{jlr}}\right\}\delta \;\left(g_{\mathrm{il}},g_{\mathrm{jr}}\right),$$

where 1 is the number of layers in the multilayer network, *A*_*ijl*_ is the functional connectivity matrix, *P*_*ijl*_ is the corresponding null model matrix (i.e., Newman–Girvan null model) defined as the *k*_*il*_ × *k*_*jl*_/2*m*_*l*_, where *m* is the average edge weight in the matrix, *g*_*il*_ gives the community assignment of node *i* in layer *l*, and *g*_*jr*_ gives the community assignment of node *j* in layer *r*. The *γ* is the *structural resolution parameter* which defines the weight of intralayer connections and thus the number of obtained modules, while *ω* is the *temporal resolution parameter* which sets the weight of the interlayer edges that link each node *i* to itself across layers. When the value of *γ* is small, the maximization of Q produces relatively large communities, while large values result in more communities with smaller number of nodes. Given that the value of *ω* defines the consistency of multilayer modules, large values relative to intralayer edges result in communities that are more similar to one another across layers. We set these parameters to frequently used default values of *γ* = 1, ω = 1 (Betzel et al., 2017; Telesford et al., 2017). Additional analyses with varying parameters around these default values can be found in the supplementary material. As the multilayer community detection algorithm is stochastic, the modularity index (i.e., Q) was averaged across 100 optimizations of the modularity quality function. The multilayer modularity analysis was implemented with code from Jeub et al (Lucas G. S. Jeub, Marya Bazzi, Inderjit S. Jutla, and Peter J. Mucha, “A generalized Louvain method for community detection implemented in MATLAB,” http://netwiki.amath.unc.edu/GenLouvain (2011–2017).

The algorithm was used with the randomization option “moverandw” instead of the default “move” option, as this has been shown to mitigate some undesirable behavior for multilayer modularity with ordinal coupling (Bazzi et al., 2016).

### 2.7 Multilayer metrics

In order to describe the temporal variability of community (i.e., modular) structure, we calculated the *node flexibility* score which represents the number of times that a node switches communities over time, normalized by the total possible number of switches (Bassett et al., 2011). We obtained the global flexibility scores by averaging over all brain regions included in the analysis.

To better understand the underlying mechanism of the network flexibility over time, we calculated additional three measures: *node promiscuity* (Papadopoulos, Puckett, Daniels, & Bassett, 2016), *node cohesion strength*, and *node disjointedness* (Telesford et al., 2017).

In order to investigate the tendency of brain regions to change allegiance between limited or multiple (i.e., temporal integration) communities, we calculated the *node promiscuity* which reflects the fraction of all networks in which the node participates at least once, across all network layers (Papadopoulos et al., 2016). The global promiscuity was defined as the average promiscuity over all nodes.

Node cohesion strength and node disjointedness quantify the node changes based on mutual versus independent changes, respectively (Telesford et al., 2017).

Node cohesion strength is defined as a cohesion matrix, where the edges represent the number of times a pair of nodes change to the same community together. Cohesion strength of a node is the sum of its row values in the cohesion matrix, with higher values indicative of frequent changes with other nodes, and lower values implying infrequent changes with other nodes. Node disjointedness is defined by the number of times a node switches communities independently of other nodes, divided by the number of times a node can change communities. Higher values indicate frequent independent changes, and lower values imply infrequent switches of communities of a given node independently of other nodes. The calculated metrics were averaged over all brain regions in order to obtain the global indices of disjoint and cohesive changes of a brain network across time.

To understand, how the present dataset corresponds to known functional networks, we compared the community partitions obtained on this data to the seven predefined resting state networks (Schlesinger et al., 2017): FPCN, DMN, DAN, SVAN, LIMB, SM, and VIS.

First, the cooccurrence of brain regions was summarized with a *module allegiance matrix*, where the *ij*th element present the percentage of time points in which both region *i* and region *j* belong to the same community (Mattar, Cole, Thompson‐Schill, & Bassett, 2015). Thus, regions that are consistently grouped together in the same network, across time, have high allegiance values.

Then, using the module allegiance matrix, we computed the *dynamic recruitment of a network*, that estimated the probability that its regions cooccur in predefined networks with regions from the same network across time points (Mattar et al., 2015). The recruitment coefficient was averaged over all brain regions in order to obtain the global dynamic recruitment.

In addition, all of the calculated multilayer metrics were summarized across predefined networks (Schlesinger et al., 2017); to further explore the significance of the obtained results in the context of well‐known resting state networks.

As previously stated, the multilayer community detection algorithm is stochastic and therefore the obtained measures were averaged across 100 optimizations of the modularity quality function.

To provide better intuitive understanding of these metrics, Telesford et al. (2017) gave the following example which relates splitting versus merging communities to the measures of disjointedness and cohesion (for VIS representation, please see Telesford et al., 2017). If a community splits in two, the cohesion is nonzero, as a subgroup of nodes from an old Community A moves to a new Community B, and a separate group of nodes moves from the old Community A to a new Community C. Node disjointedness is in this case zero, as individual nodes did not switch communities independently of other nodes. The same is true for merging communities, as we once again have high cohesion and zero node disjointedness.

The calculation of global flexibility, cohesion strength, and disjointedness was implemented with code from the Network Community Toolbox (http://commdetect.weebly.com/).

### 2.8 Null models

To determine the statistical significance of the temporal evolution of functional brain networks and to test against the null hypothesis that there is no smooth reconfiguration between consecutive time points, we compared the real functional networks to a *temporal null model*. This null model was created by shuffling the time layers in the multilayer network uniformly at random across time (Chai, Mattar, Blank, Fedorenko, & Bassett, 2016; Sizemore & Bassett et al., 2017). Importantly, the temporal null model constructed in such a way, preserves connectivity within a network layer but eliminates the dependencies between layers over time.

Further, we computed a *nodal null model* to contrast the network recruitment coefficient obtained on our data against the null hypothesis that roles of the regions in the network are identical (i.e., no functional subnetworks). Hence, we ensure that the community structure we calculate is not random but instead captures the modular organization of functional connectivity. This null model, similar to the configuration model for static graphs, was constructed by randomly rewiring edges occurring at the same point (Sizemore & Bassett et al., 2018).

Therefore, we computed 50 null models (temporal and nodal) for each subject's multilayer functional matrix and then optimized the multilayer modularity quality function 100 times on each of these null model networks.

The real networks were compared to null networks using Welch's two‐sample *t* test implemented in R (v. 3.5.2; The R Project for Statistical Computing; http://www.R-project.org/).

The significance level of *p*‐values was adjusted to *p* < .05/*n*, where *n* represents the number of multilayer measures that were compared between the observed and null networks.

Further, we calculated the Cohen's d *index of* effect size using an R‐based package lsr (v. 0.5), and interpreted it as follows: *d* ≥ 0.2 was considered a “small” effect size, *d ≥* 0.5 represented a “medium” effect size and *d* ≥ 0.8 a “large” effect size (Cohen, 1988).

### 2.9 Statistical analysis

#### 2.9.1 Brain modular reconfiguration and age at baseline

To test the hypothesis that the brain modular reconfiguration is related to aging, we performed multiple linear regression analysis (*lme4* package [v. 1.1‐18‐1] in R [v. 3.5.2]), in which the outcome was a particular multilayer measure and the predictor was age at baseline (grand‐mean‐centered variable). Gender (female = 1, male = 0) and education (on a scale from 1 to 3; 1 = high school with or without vocational education, 2 = higher education entrance qualification, business school or university of applied sciences, or 3 = university degree) were entered as nuisance covariates into the model, as previous studies have related these variables to brain's functional network organization (Chan et al., 2018). Further, we included motion as an additional nuisance covariate, defined as the average framewise displacement (FD) across four measurement occasions, as head motion has been shown to have an important impact on brain network topology (De Vico Fallani, Richiardi, Chavez, & Achard, 2014).

Regression models were calculated for each of the global and network‐specific (i.e., FPCN, DMN, DAN, SVAN, LIMB, SM, VIS) multilayer measures (global flexibility, promiscuity, cohesion strength, disjointedness, and recruitment).

The effect sizes (i.e., partial *η* ^2^) were calculated using the *lmSupport* package (v. 2.9.13) in R (v. 3.5.2). The significance level of *p*‐values was adjusted to *p* < .05/*n*, where *n* represent the number of models tested.

In addition, to investigate the relationship between the metrics, Pearson's correlation analysis was used to calculate the association between the modularity index (Q), global recruitment, and global flexibility. The analysis was performed using an R‐based package called *psycho* (v. 0.4.0.). In addition, partial correlation analysis was conducted to test the relationship between these metrics while controlling for the effects of age at baseline using the *ppcor* (v. 1.1) package in R.

The results were visualized using *sjPlot* (v. 2.6.1) and *ggplot* (v. 2‐3.0.0) packages in R.

#### 2.9.2 Longitudinal change in cognitive performance

We performed linear mixed effects (LME) analysis (*lme4* package (v.1.1‐18‐1) in R (v. 3.5.2); Bates, Mächler, Bolker, & Walker, 2015) to assess the longitudinal change in cognitive performance (i.e., processing speed, learning/memory encoding). As fixed effects, we entered time, age at baseline (grand‐mean‐centered variable), and their interaction term into the model. As random effects, we had intercepts for subjects as well as by‐subject random slopes for the effect of time. Gender and education were entered as nuisance covariates.

In addition, as it is very likely that the change in cognitive performance between the baseline and the 1‐year follow‐up assessment is influenced by the increased familiarity with the testing situation (Hoffman, Hofer, & Sliwinski, 2011), we added a “retest effect” (baseline = 0, 1‐year follow‐up = 1, 2‐year follow‐up = 1, 4‐year follow‐up = 1) as a covariate in the LME models.

Linear mixed models were fit by maximum likelihood and the p‐values were obtained from the t‐statistic using Satterthwaites's approximation to the denominator degrees of freedom (*lmerTest* package (v. 3.0‐1) in R (v. 3.5.2); Kuznetsova, Brockhoff, & Christensen, 2017). The mixed models were fitted separately for each of the cognitive domains. The significance level of *p*‐values was adjusted to .05/*n*, where *n* represents the number of cognitive domains.

The results were visualized using the *ggplot* (v. 2‐3.0.0) package in R.

#### 2.9.3 Brain‐cognition association

To investigate the “change–change” brain‐cognition association, we performed Pearson's correlation analysis (R‐based package *psycho* (v. 0.4.0.)) between the individual rate of change in cognition and the multilayer measures (i.e., global flexibility) which summarize network properties across time. In addition, partial correlation analysis was done to test the brain‐cognition relationship while controlling for the effects of age at baseline using the *ppcor* (v. 1.1) package in R.

The individual rate of change in cognitive performance, defined as the subject‐specific slope of the regression line between time and the cognitive scores, was derived from the LME models described in the previous section (Ng et al., 2016, 2018). The results were visualized using the *ggplot* (v. 2‐3.0.0) package in R.

## 3 RESULTS

### 3.1 Longitudinal reconfiguration of functional modules

We used the modularity maximization algorithm to define the temporal communities in the multilayer functional connectivity matrix (distributions of the number of modules are plotted in Supplementary Figure S1).

**FIGURE 1:**
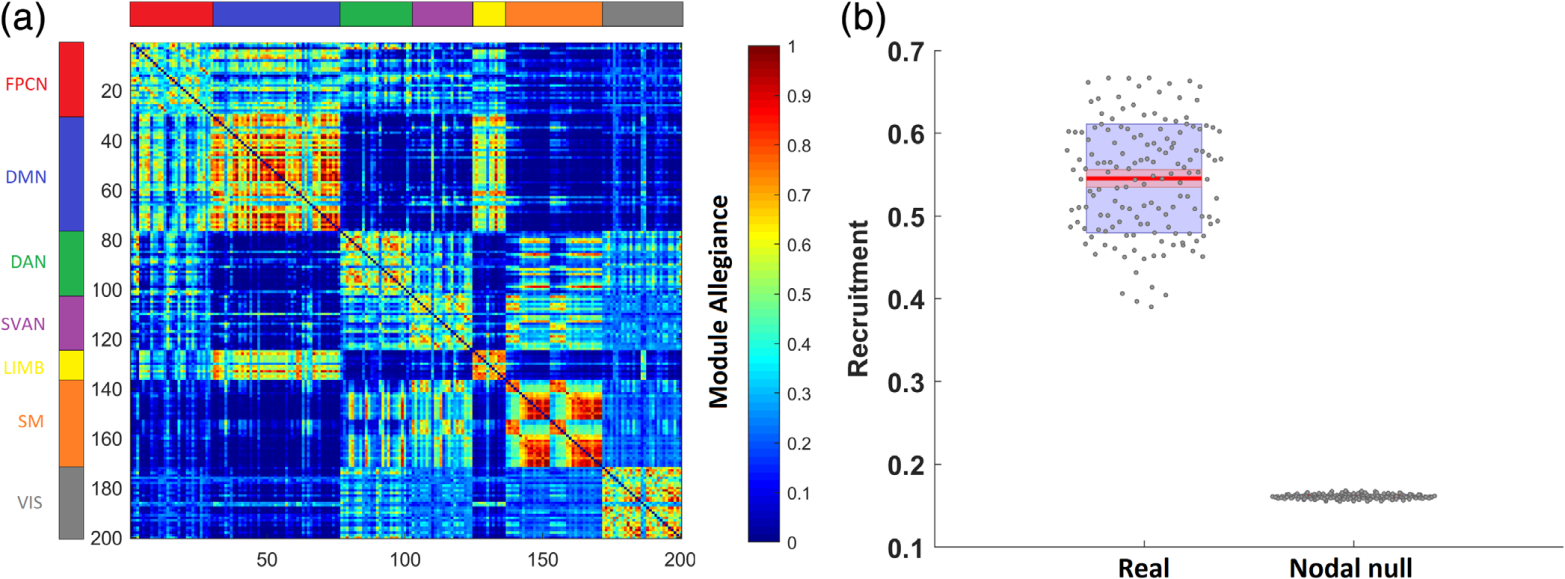
(a) The module allegiance matrix represents the probability that two brain regions are part of the same community across the 4‐year interval. The brain regions are ordered according to the predefined network they belong to. Higher values along the diagonal of the matrix suggest that networks from the Schaefer atlas tend to be recruited together in the same communities across the 4‐year interval. (b) Comparison of global recruitment of real networks to a nodal null model. The center red lines represent the mean, and the light red bars and light blue bars represent 95% confidence interval and *SD*, respectively. This figure was generated using notBoxPlot (https://github.com/raacampbell/notBoxPlot)

First, we calculated the dynamic recruitment coefficient, which quantifies the probability that nodes of a network are consistently assigned to the same module across different time layers. Then, we compared the observed global recruitment to that of a nodal null model, to ensure that the community structure we obtained is not random but instead captures the organization of well‐known resting state networks. The module allegiance matrix in Figure 1a provides a summary representation of how brain regions and networks are dynamically engaged across four time points.

The observed global recruitment coefficient was significantly higher than in the nodal null network (*t*(298) = 71.5, *p* < .0001, *d* = 8.26) (Figure 1b). This result suggests that the regions tend to be recruited to their own networks and are less integrated with other networks across time, suggesting that the community structure we obtained is not random but represents meaningful modular organization. Self‐recruitment scores for each of the networks can be found in Supplementary Figure S2.

Further, we calculated several temporal measures in order to characterize the patterns of modular change across time, which we then compared to a temporal null model to determine whether the obtained values were higher or lower than expected.

We investigated the longitudinal network reconfiguration by calculating the flexibility score, which indicates the number of times nodes switch their community assignment across temporal layers, and global promiscuity, which is defined as the fraction of all communities in which the node participates at least once, across all network layers.

**FIGURE 2:**
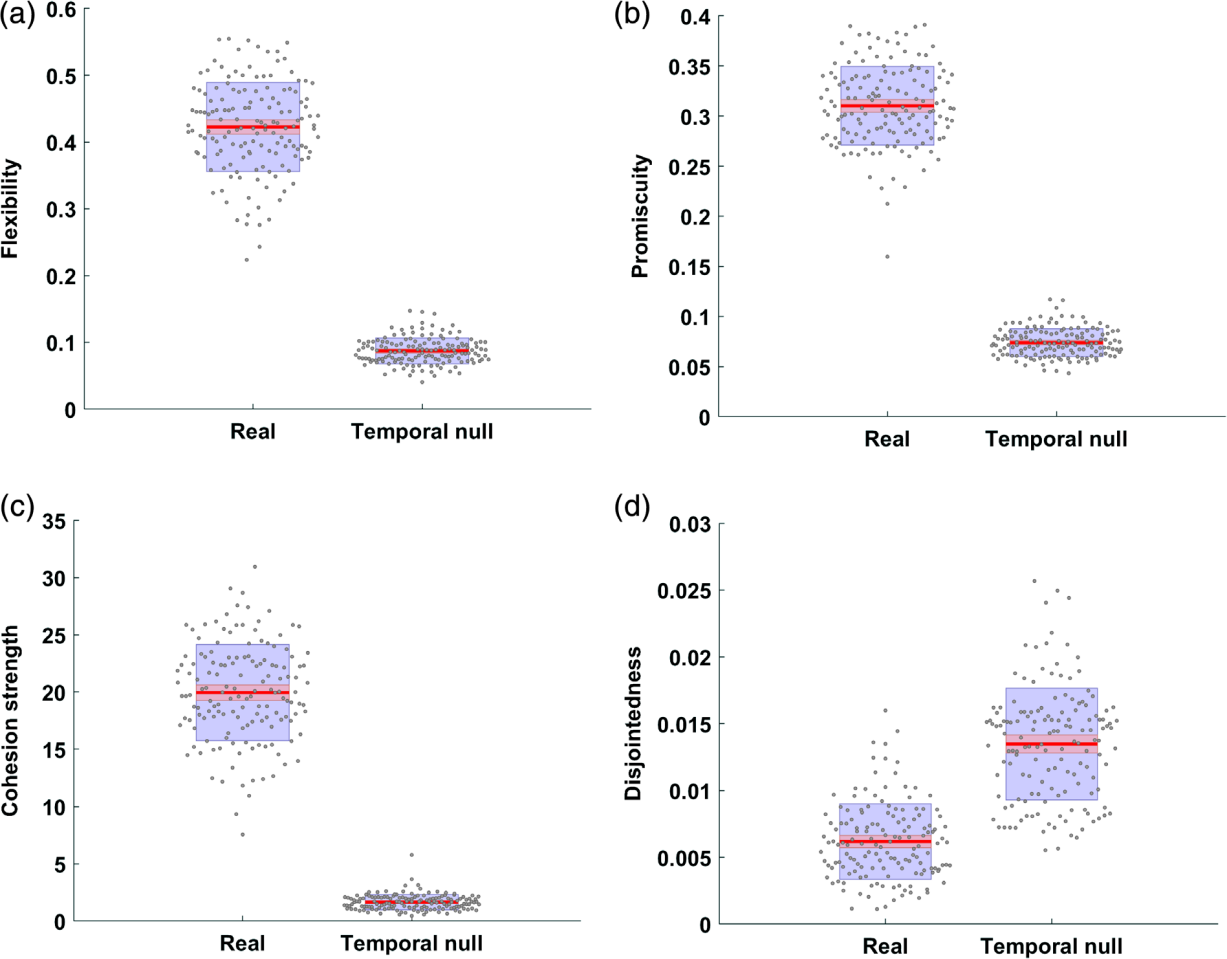
Comparison of global flexibility (a), promiscuity (b), cohesion strength (c), and disjointedness (d) of real networks to a temporal null model. The center red lines represent the mean, and the light red bars and light blue bars represent 95% confidence interval and *SD*, respectively. This figure was generated using notBoxPlot (https://github.com/raacampbell/notBoxPlot)

The observed global flexibility (*t*(298) = 59.2, *p* < .0001, *d* = 6.84), and global promiscuity (*t*(298) = 69.6, *p* < .0001, *d* = 8.04), were significantly higher than in the temporal null model suggesting that the functional brain displayed more change than expected in a temporal null network (Figure 2a,b).

Further, to better understand the underlying mechanism of brain flexibility over time, we calculated another two measures: global cohesion strength and disjointedness.

The observed cohesion strength (*t*(298) = 52.7, *p* < .0001, *d* = 6.09) was significantly higher than in the temporal null model, suggesting that the observed functional networks had a greater range of community dynamics in comparison to the null network (Figure 2c).

However, the observed disjointedness (*t*(298) = −17.7, *p* < .0001, *d* = 2.05) was significantly lower than in the null model, suggesting that the change in community structure was driven by subgroups of nodes switching communities (indicated by a higher cohesion strength) instead of individual nodes switching independently of other nodes (Figure 2d).

All statistically significant effects survived multiple comparison corrections (*p* < .05 corrected for five measures).

Finally, Supplementary Figure S4 shows that our results were robust in both unthresholded and thresholded matrices.

### 3.2 Negative correlation between modularity and flexibility

Next, we wanted to assess the relationship between flexibility and the quality of modular decomposition. In order to do so, we used the modularity index Q, describing the quality of modular structure across the 4‐year interval. Higher values of Q indicate better modular definition of a given functional network, while lower values suggest lower segregation between networks and thus worse modular decomposition. Further, we explored the association between flexibility and recruitment to test if the consistency to which nodes are assigned to the same module across time is related to the frequency of nodes switching between modules.

**FIGURE 3:**
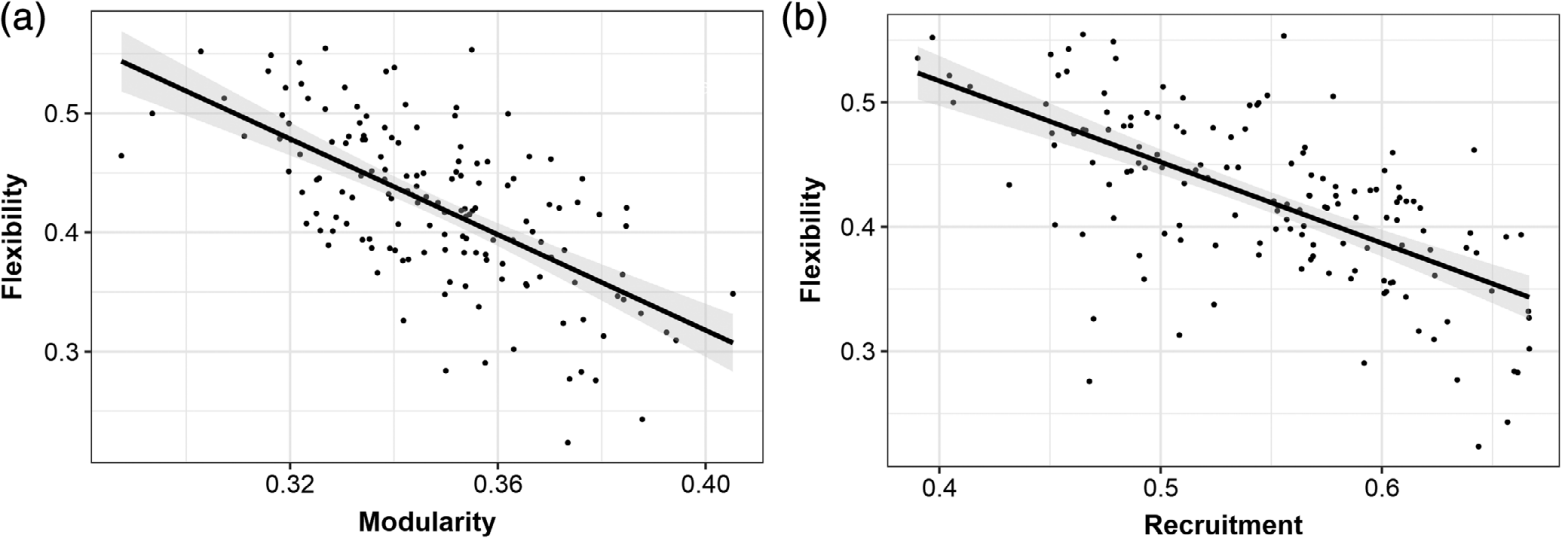
The correlation analysis showed a strong negative association between modularity and flexibility (*r*(148) = −.64, *p* < .0001), and global recruitment and flexibility (*r*(148) = −.64, *p* < .0001)

Pearson's correlation analysis showed strong negative correlation between modularity and global flexibility (*r*(148) = −.64, 95% CI [−0.72, −0.53], *p* < .0001), implying that more segregated networks are also more resistant to change, and thus exhibit lower variability across a given time span (Figure 3a). Also, there was a strong negative correlation between global recruitment and flexibility (*r*(148) = −.64, 95% CI [−0.73, −0.54], *p* < .0001), suggesting that flexibility is related to nodes not being consistently assigned to the same module across time (Figure 3b).

In addition, partial correlation analysis was conducted to test the relationship between modularity, recruitment and flexibility while controlling for the effects of age at baseline. The association between modularity and flexibility (*r*(148) = −.62, 95% CI [−0.71, −0.51], *p* < .0001), and recruitment and flexibility (*r*(148) = −.63, 95% CI [−0.71, −0.52], *p* < .0001) remained significant after controlling for age at baseline.

### 3.3 The role of age at baseline in modular reconfiguration

**FIGURE 4:**
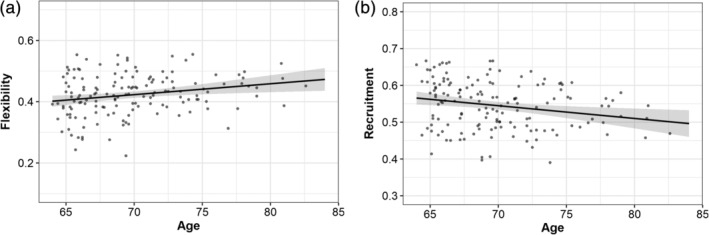
Global flexibility and recruitment in association with age at baseline. The results indicate greater global flexibility and lower global recruitment across time in older participants

We were interested in assessing if multilayer measures were related to age at baseline in our sample of elderly subjects (Figure 4).

Multiple regression analysis showed higher flexibility in older participants, indicating higher variability in network structure (*b* = .0036, *p* = .006, partial *η* ^2^ = 0.0504; Table 1).

**TABLE 1:** Association between multilayer measures and age at baseline in multiple regression models. Statistically significant effects (*p* < .05) appear in bold

Metric	Predictors	Estimates	*SE*	CI	*p*	Partial *η* ^2^
*Flexibility*	(Intercept)	0.4165	0.0200	0.3773–0.4557	**<.001**	**0.7495**
	Gender	−0.0003	0.0109	−0.0218 to 0.0211	.975	<0.0001
	Education	0.0075	0.0068	−0.0059 to 0.0208	.275	0.0082
	Age	0.0036	0.0013	0.0010–0.0061	**.006** ^a^	**0.0504**
	FD	−0.0480	0.0509	−0.1478 to 0.0517	.347	0.0061
*Promiscuity*	(Intercept)	0.2964	0.0120	0.2728–0.3201	**<.001**	**0.8084**
	Gender	0.0099	0.0066	−0.0030 to 0.0229	.132	0.0156
	Education	0.0051	0.0041	−0.0030 to 0.0131	.218	0.0104
	Age	0.0008	0.0008	−0.0007 to 0.0024	.277	0.0081
	FD	−0.0111	0.0305	−0.0714 to 0.0492	.716	0.0009
*Cohesion strength*	(Intercept)	18.5624	1.2832	16.0473–21.0774	**<.001**	**0.5907**
	Gender	1.1675	0.7021	−0.2087 to 2.5436	.099	0.0187
	Education	0.3476	0.4379	−0.5106 to 1.2057	.429	0.0043
	Age	0.0845	0.0822	−0.0766 to 0.2457	.306	0.0072
	FD	0.1038	3.2663	−6.2981 to 6.5057	.975	<0.0001
*Disjointedness*	(Intercept)	0.0068	0.0009	0.0051–0.0085	**<.001**	**0.3037**
	Gender	−0.0010	0.0005	−0.0019 to −0.0001	**.034**	**0.0306**
	Education	0.0001	0.0003	−0.0005 to 0.0007	.782	0.0005
	Age	0.0001	0.0001	−0.0000 to 0.0002	.182	0.0123
	FD	−0.0014	0.0022	−0.0057 to 0.0028	.512	0.0030
*Recruitment*	(Intercept)	0.5276	0.0196	0.4890–0.5663	**<.001**	**0.8338**
	Gender	0.0142	0.0107	−0.0069 to 0.0354	.186	0.0120
	Education	−0.0027	0.0067	−0.0159 to 0.0105	.689	0.0011
	Age	−0.0035	0.0013	−0.0059to 0.0010	**.006** ^a^	**0.0501**
	FD	0.0738	0.0498	−0.0246 to 0.1722	.140	0.0149

*Note:* FD—average framewise displacement across four time points.

^a^ Survives multiple comparison correction (*p* < .05 corrected for five measures).

There were no significant age effects on global promiscuity (*b* = .0008, *p* = .277, partial *η* ^2^ = 0.0081), suggesting that there were no age differences in the number of networks the nodes switch between (Table 1).

We did not observe any significant association between the global cohesion strength (*b* = .0845, *p* = .306, partial *η* ^2^ = 0.0072) or disjointedness (*b* = .0001, *p* = .182, partial *η* ^2^ = 0.0123) and age at baseline, implying that the extent of mutual or independent changes in community structure (over the 4‐year time interval), was not linked to age at baseline.

These results suggest that although there are age differences in the extent of global flexibility, the mechanisms of this change were not significantly related to age and thus homogeneous across the sample.

Next, the global recruitment was significantly negatively associated with age at baseline (*b* = −.0035, *p* = .006, partial *η* ^2^ = 0.0501), suggesting a lower probability of a given region to be grouped in the same community as other regions of its own community across time in older age (Table 1). This result points to higher instability in modular organization and network reconfiguration with advancing age.

Motion and education were not significantly related to any of the metrics.

Importantly, the associations between age at baseline and flexibility survived multiple comparison correction (*p* < .05, corrected for five models).

Results of linear models including flexibility (the only metric significantly associated with age) calculated with other values of multilayer parameters (*γ* and *ω* = 0.9–1.5, increments of 0.1) can be found in Supplementary Figure S5. Despite some variability across a range of multilayer parameters, supplementary results were largely consistent with the main findings. The nonsignificant association between flexibility and age at baseline was situated around high values of *ω* (interlayer coupling parameter). Higher values of *ω* increase the similarity of layers across time thus reducing variance in temporal modular structure uniformly across subjects. However, it is also possible that age‐related flexibility changes manifest scale‐specific patterns, as suggested elsewhere (Betzel et al., 2015; Betzel et al., 2019), but given that there are currently no standards regarding the choice of parameter values, this research question falls outside the scope of this paper.

**FIGURE 5:**
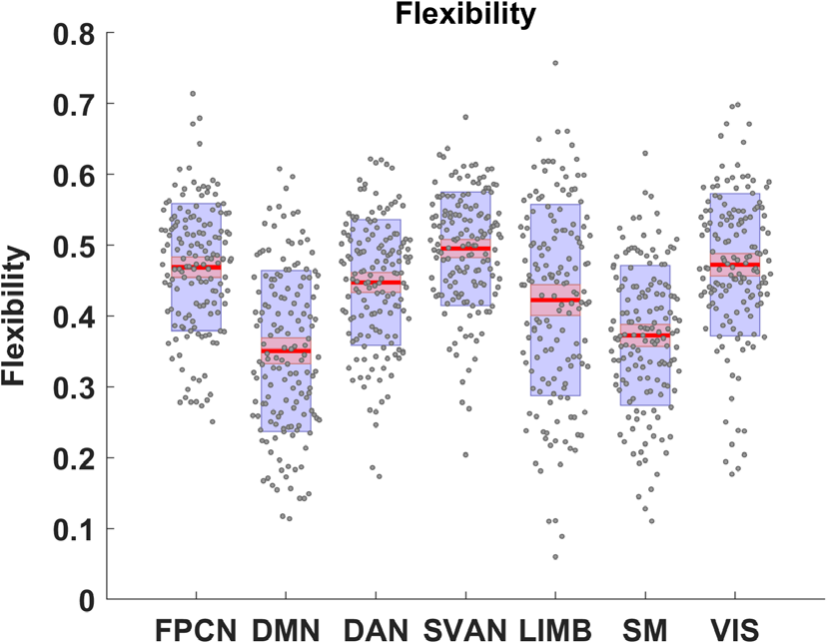
Visualization of summary statistics for network‐specific flexibility across the 4‐year interval. The center red lines represent the mean, and the light red bars and light blue bars represent 95% confidence interval and *SD*, respectively. This figure was generated using notBoxPlot (https://github.com/raacampbell/notBoxPlot)

Next, we computed the flexibility metric for predefined resting state networks (Schlesinger et al., 2017)—FPCN, DMN, DAN, SVAN, LIMB, SM, and VIS (Figure 5). The SVAN network had the highest mean flexibility, while the DMN network had the lowest mean flexibility across all subjects (Figure 5). All networks had considerable between‐subject variability, but the LIMB network showed the highest *SD* and the widest range of flexibility values across all subjects within our sample (Figure 5). This intersubject variance in LIMB regions possibly reflects the relatively small number of regions within this network (i.e., 12 brain regions).

**FIGURE 6:**
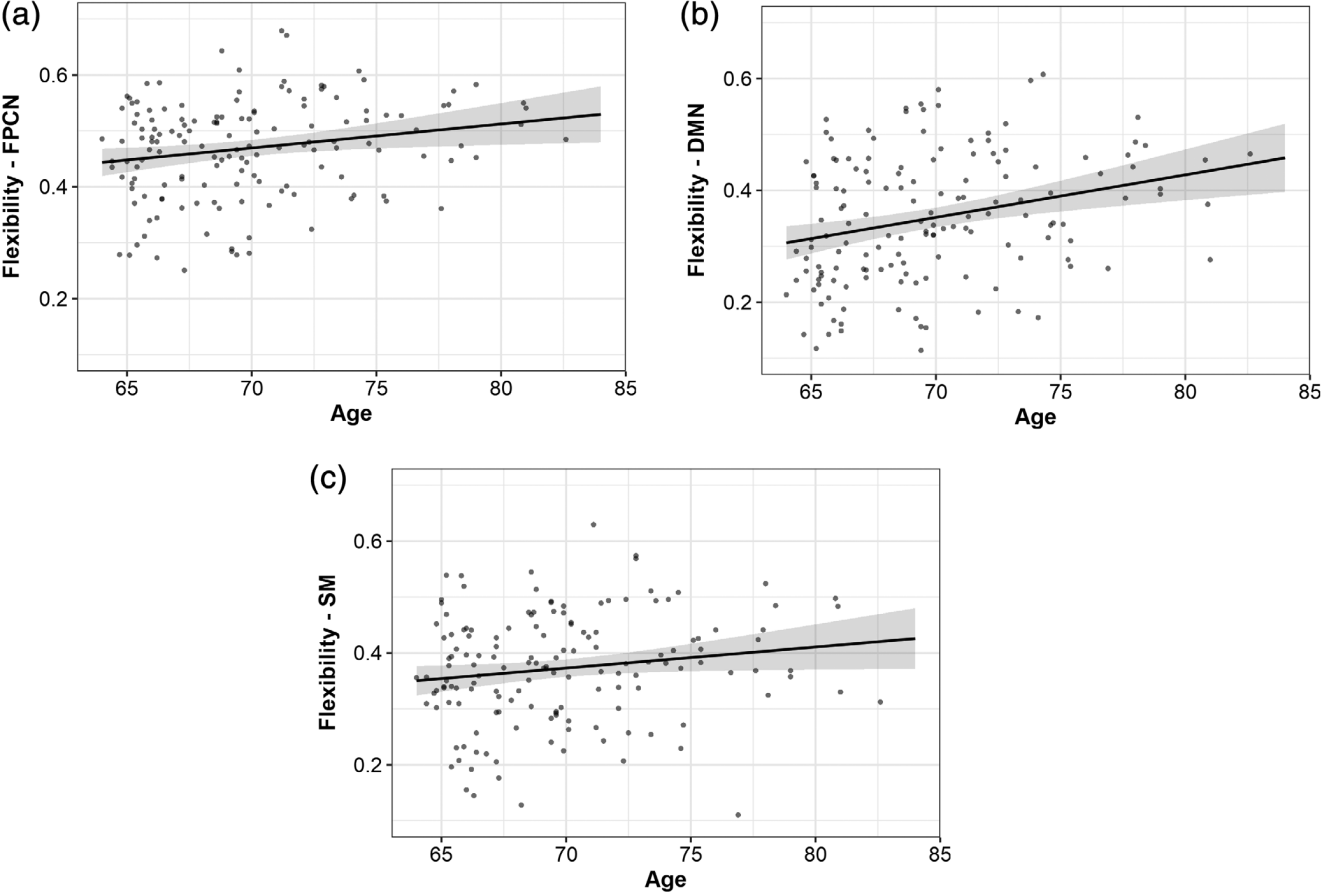
Network‐specific flexibility in association with age at baseline. The results indicate greater modular flexibility of the frontoparietal control, default mode, and somatomotor networks across time in older participants

As with the global measures, we ran multiple regression models to assess the relationship between network‐specific flexibility and age at baseline (Table 2). Interestingly, the flexibility of DMN (*b* = .0076, *p* < .001, partial *η* ^2^ = 0.0824); FPCN (*b* = .0043, *p* = .014, partial *η* ^2^ = 0.0407); and SM (*b* = .0038, *p* = .047, partial *η* ^2^ = 0.0269) was higher with older age, suggesting that older subjects have higher flexibility in these networks (Figure 6).

**TABLE 2:** Association between network‐specific flexibility and age at baseline in multiple regression models. Statistically significant effects (*p* < .05) appear in bold

Network	Predictors	Estimates	*SE*	CI	*p*	Partial *η* ^2^
*FPCN*	(Intercept)	0.4360	0.0270	0.3831–0.4889	**<.001**	**0.6426**
	Gender	0.0186	0.0148	−0.0104 to 0.0475	.211	0.0108
	Education	0.0130	0.0092	−0.0050 to 0.0311	.160	0.0136
	Age	0.0043	0.0017	0.0009–0.0077	**.014**	**0.0407**
	FD	−0.0269	0.0687	−0.1617 to 0.1078	.696	0.0011
*DMN*	(Intercept)	0.3614	0.0329	0.2969–0.4258	**<.001**	**0.4545**
	Gender	−0.0411	0.0180	−0.0764 to −0.0058	**.024**	**0.0348**
	Education	0.0114	0.0112	−0.0106 to 0.0333	.313	0.0070
	Age	0.0076	0.0021	0.0035–0.0117	**<.001** ^a^	**0.0824**
	FD	−0.0765	0.0837	−0.2405 to 0.0875	.362	0.0057
*SM*	(Intercept)	0.3470	0.0293	0.2895–0.4045	**<.001**	**0.4910**
	Gender	0.0471	0.0161	0.0157–0.0786	**.004** ^a^	**0.0561**
	Education	0.0019	0.0100	−0.0178 to 0.0215	.852	0.0002
	Age	0.0038	0.0019	0.0001–0.0074	**.047**	**0.0269**
	FD	−0.0056	0.0747	−0.1520 to 0.1407	.940	<0.0001

*Note:* FD—average framewise displacement across four time points.

Abbreviations: DMN, default mode network; FPCN, frontoparietal control network; SM, somatomotor network.

^a^ Survives multiple comparison correction (*p* < .05 corrected for seven networks).

Linear models with remaining networks did not show any significant association between age at baseline and network flexibility (Supplementary Table S2).

Of all of the tested models, only the association between age and DMN flexibility survived multiple comparison correction (*p* < .05, corrected for seven networks).

Finally, we wanted to explore the mechanisms of flexibility for the networks that had significant effects of age at baseline; therefore, we computed linear regression models with network‐specific promiscuity and recruitment for the DMN, FPCN, and SM networks.

**FIGURE 7:**
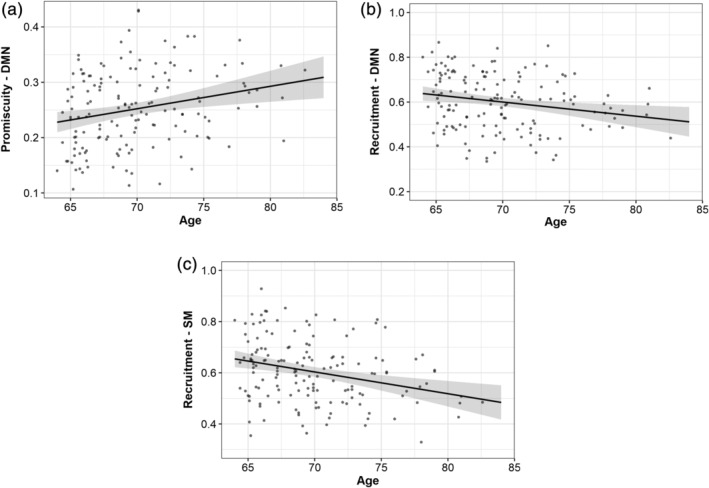
Network‐specific promiscuity and recruitment in association with age at baseline. The results indicate greater modular promiscuity of the default mode and lower self‐recruitment of the default mode and somatomotor networks across time in older participants

The results showed higher DMN promiscuity (*b* = .0041, *p* = .002, partial *η* ^2^ = 0.0627) with older age (Figure 7a, Supplementary Table S3). This can be interpreted as a higher tendency of brain regions belonging to the DMN to segregate from this network and connect to all other networks across the whole brain.

Not surprisingly, the self‐recruitment of the DMN (*b* = −.0063, *p* = .006, partial *η* ^2^ = 0.0501) and SM (*b* = −.0085, *p* < .001, partial *η*2 = 0.0851) was lower with higher age (Figure 7b,c, Supplementary Table S3), suggesting that the network flexibility across the 4‐year interval was related to lower probability of brain regions from these networks to be categorized into same communities as other regions from the corresponding networks.

In Supplementary Figure S6, in which the module allegiance matrix was calculated for the youngest 10% and for the oldest 10% of the sample, we can see that modules, and especially the DMN, tend to be more consistently recruited across time points in younger in comparison to older subjects.

All statistically significant effects survived multiple comparison corrections (*p* < .05 corrected for six tests).

### 3.4 The role of modular reconfiguration in cognitive performance

First, we performed LME analysis to investigate longitudinal change in cognitive functioning (Supplementary Figure S7). We found a statistically significant decline in processing speed (*b* = −.5276, *p* < .001) and learning/memory encoding (*b* = −.5001, *p* = .003). Older age was associated with lower performance in both domains (Table 3). Finally, there was a significant interaction between age at baseline and time (*b* = −.0512, *p* = .029), suggesting that older participants had a more significant decline in processing speed across the 4‐year time interval.

**TABLE 3:** Longitudinal (time) and cross‐sectional effects (age at baseline) in the LMEs models of cognitive performance. Statistically significant effects (*p* < .05) appear in bold

	Predictors	Estimates	*SE*	CI	*p*
*Processing speed*	Retest	1.7722	0.4029	0.9826–2.5619	**<.001** ^a^
	Gender	−0.7603	1.1677	−3.0490 to 1.5284	.516
	Education	0.8785	0.7134	−0.5197 to 2.2768	.220
	Time	−0.5276	0.1271	−0.7766 to −0.2785	**<.001** ^a^
	Age	−0.7564	0.1430	−1.0366 to −0.4762	**<.001** ^a^
	Time × age	−0.0512	0.0232	−0.0967 to −0.0057	**.029**
*Learning/memory encoding*	Retest	0.3933	0.5441	−0.6730 to 1.4596	.470
	Gender	2.0082	1.1305	−0.2076 to 4.2240	.078
	Education	2.1896	0.6893	0.8387–3.5406	**.002** ^a^
	Time	−0.5001	0.1648	−0.8231to −0.1771	**.003** ^a^
	Age	−0.5589	0.1365	−0.8264 to −0.2914	**<.001** ^a^
	Time × age	−0.0306	0.0298	−0.0890 to 0.0277	.304

Abbreviation: LME, linear mixed effect.

^a^ Survives multiple comparison correction (*p* < .05 corrected for two measures).

Also, participants with better education had higher scores in learning/memory encoding (*b* = 2.1896, *p* = .002). In addition, there was a significant retest effect on processing speed (*b* = 1.7722, *p* < .001), but not on learning/memory encoding (*b* = .3933, *p* = .470).

All statistically significant effects (except the interaction effect on processing speed) survived multiple comparison corrections (*p* < .05 corrected for two domains).

Next, we conducted Pearson's correlation analysis to assess the association between flexibility and change in cognitive performance. We did not find a significant relationship between change in processing speed and global flexibility (*r*(148) = −.03, 95% CI [−0.19, 0.13], *p* = .74). This was also true for the association between learning/memory encoding and global flexibility (*r*(146) = −.008, 95% CI [−0.17, 0.15], *p* = .92).

Finally, the brain‐cognition association was also tested for network‐specific flexibility, however, there were no significant relationships found between any of the networks (i.e., FPCN, DMN, DAN, SVAN, LIMB, SM, VIS) and change in processing speed or learning/memory encoding (see Supplementary material for further details).

## 4 DISCUSSION

In the present study, we investigated the temporal change of the brain's modular structure in healthy aging. We applied the multilayer model, which resulted in several measures summarizing the characteristics of longitudinal network reconfiguration in healthy elderly.

We showed significantly higher variability and a greater range of modular “dynamics” over the course of 4 years in older subjects' functional networks in comparison to temporal null networks. Further, flexibility was significantly associated with age at baseline, with older participants having higher global and network‐specific flexibility, particularly evident in the FPCN, DMN, and SVAN.

We also observed a decrease in the global network recruitment with older age, indicating community structure reorganization in which some brain regions are inconsistently assigned to their modules across different time points. This was most evident for the DMN which had lower time‐dependent self‐recruitment in older participants.

Over the same 4‐year time interval, we observed a significant decrease in processing speed and learning/memory encoding (a finding which is well in line with previous cognitive aging studies (Ng et al., 2016; Salthouse, 2010; Staffaroni et al., 2018). However, the decline in cognitive performance was not related to the multilayer brain dynamics, implying an absence of simultaneous (i.e., change–change) relations between changes in functional brain network organization and cognition measures.

The multilayer modularity approach provides several advantages in comparison to more traditional methods for community detection. Similar to “single‐layer” modularity, it does not require a predefined set of networks, it is completely data‐driven (de Domenico, 2017) and applicable to an individual level (Shine et al., 2016). However, in contrast to “single‐layer” modularity, it partitions all temporal layers simultaneously, maintaining a consistent set of modules across all layers, thus ensuring the same definition of networks across all time points (Mucha et al., 2010).

To our knowledge, this is the first study with the application of multilayer community detection on longitudinally acquired data in healthy elderly. Nonetheless, our findings are in line with previous studies, suggesting unstable network architecture and substantial functional reconfiguration with aging.

Interestingly, global flexibility was highly negatively correlated with modularity, suggesting that more segregated networks are also more resistant to change, and thus exhibit lower variability across a certain time span (Harlalka, Bapi, Vinod, & Roy, 2019; Meunier, Lambiotte, & Bullmore, 2010; Ramos‐Nuñez et al., 2017).

We also found a highly significant negative correlation between global flexibility and recruitment, which implied that higher network flexibility is related to a more random nature of brain dynamics in which functional modules are not persistently recruited across time.

Modular brain networks exhibit a fine balance of dense within‐network connections and sparse connections between regions in networks with different processing roles (Meunier et al., 2010).

In our recent study, including the present sample (but also encompassing participants with missing data at some time points), we explored changes in the functional segregation of resting state networks across time (Malagurski et al., 2020). We showed a decrease over a 4‐year interval in the functional segregation of associative networks, including the default mode, FPCN and SVAN networks. Thus, it is possible that a loss of within‐network integrity might have resulted in nodes grouping in fewer and larger modules, and at the same time losing functional segregation and seeing more nodal movement between modules across the 4‐year time interval (Schlesinger et al., 2017).

These findings are in line with other research that suggested increased modular variability or heterogeneity within higher order cortices in healthy elderly, indicating that the brain reconfigures during the aging process and varying cognitive demands (Peraza et al., 2018; Schlesinger et al., 2017). Furthermore, another study showed less similarity of network partitions in older healthy subjects, both as a group and across time, compared to younger participants, which implied reduced stability of network organization with aging (Iordan et al., 2018).

In the present study, we also calculated node promiscuity, cohesion strength, and disjointedness, and compared these metrics to temporal null models in order to better understand the underlying mechanism of brain flexibility over time. The observed cohesion strength was significantly higher than in the null network, while the disjointedness was significantly lower, implying that with aging subgroups of nodes cohesively reorganize into new modules instead of individual brain nodes switching communities independently of other nodes. Nevertheless, this reconfiguration pattern is related to less specific modules with more fluid connectivity between them and could possibly indicate compensatory reconfiguration of functional networks due to declining cognitive performance or impaired recruitment mechanisms (Sala‐Llonch, Bartrés‐Faz, & Junqué, 2015). Further, cohesion strength was not significantly associated with age at baseline, which means that the mechanism of longitudinal change in functional configuration was more uniform across the sample.

Although global promiscuity was significantly higher than in the temporal null model it was not significantly associated with age, further reinforcing the notion of a more uniform mechanism of global flexibility across the age span found in our sample.

Importantly, we found a significant relationship between flexibility and age, more specifically, older subjects tended to have higher flexibility in several predefined resting state networks, such as the FPCN, DMN, and SM networks. All networks exhibited significant levels of longitudinal flexibility, but these networks seem to present more variable change across the age range present in our sample. However, it should be noted that only the DMN flexibility—age association survived multiple comparisons correction, so the overall results should be interpreted with caution.

Moreover, given that there were some network‐specific effects on functional flexibility, we wanted to explore if the mechanism of change—promiscuity and recruitment—was diverse across age groups, despite the absence of age effects on the global average of these metrics.

Our findings pointed to higher promiscuity coupled with lower self‐recruitment of the DMN in older age, which can be interpreted as brain regions of this network being inconsistently recruited across time and less segregated from regions belonging to other communities. Lower self‐recruitment was also found for the SM network, pointing to a lower functional specialization of these networks across the 4‐year interval in healthy elderly.

The most consistent finding across studies on aging is that older adults have lower functional integrity in the DMN, compared to younger adults (Chong et al., 2019; Damoiseaux, 2017). Moreover, this network has been shown to be highly vulnerable to aging‐associated diseases such as Alzheimer's disease or cerebrovascular disease (Chong et al., 2017; Crossley et al., 2014; Kim et al., 2016).

Importantly, the relationship between aging‐related functional changes within these networks and changes in cognitive performance is not yet fully understood. In our study, we observed significantly lower processing speed and learning/memory encoding in older participants with a decline over the 4‐year time interval. However, we did not find any significant association between this reduction in cognitive performance and the multilayer measures, contrary to our expectation. Although there are no previous studies investigating longitudinal network flexibility in the context of cognitive performance, some cross‐sectional results did indicate relevant associations between modular properties and cognition in older adults (Gallen et al., 2017; Geerligs et al., 2015; Iordan et al., 2018). This lack of simultaneous relation may have been driven by some compensatory mechanism, where healthy aging individuals delay the effects of functional reconfiguration for a certain time and thus maintain cognitive ability (Reuter‐Lorenz & Park, 2014). Our study did not focus on identifying the causal role of temporal variability in cognitive functioning, but future studies should investigate lagged coupled changes in order to test if functional changes precede cognitive changes in healthy aging.

### 4.1 Methodological considerations and limitations

Although the methods applied here provide an interesting framework for investigating the longitudinal functional reconfiguration in the elderly, this approach has several methodological considerations that should be taken into account.

First, it is well‐known that the choice of nodes can significantly influence the calculation of network properties (Fornito, Zalesky, & Bullmore, 2016). We defined our nodes according to a functional atlas comprising seven resting state networks (Schaefer et al., 2018; Yeo et al., 2011), also commonly used in studies on healthy aging. Second, the number of networks in the original atlas approximated the number of modules obtained in our analysis (even though there was high interindividual variability), thus allowing more straightforward comparability between our data‐driven modules and predefined networks.

Apart from that, we only included participants with complete data (all time points) in order to maximize the overall number of temporal layers. Selective exclusion of subjects with incomplete data might introduce some bias into this type of analysis, although there was no selective attrition in our sample. As a consequence, the future research should explore different strategies for handling missing data in the context of multilayer modularity.

Finally, although multilayer metrics present a convenient method for summarizing change information across time points, this “summarization” might also obscure different rates of change existing between specific time windows, which could be more easily explored within a different statistical framework.

## 5 CONCLUSION

This study, for the first time, illustrates substantial functional network reconfiguration in healthy aging across a 4‐year time interval. In particular, the whole brain network flexibility, which reflects the tendency of brain nodes to switch between modules was significantly higher in healthy elderly than in a temporal null model and with increasing age. The modular temporal variability was not related to simultaneous changes in cognitive performance, however, further studies should include more cognitive domains or investigate lagged changes to better understand the temporal implication of the multilayer modular reconfiguration. Finally, this approach provides simple intuitive indices for overall longitudinal changes across a desired time span and it can be useful for uncovering patterns of modular variability in healthy and clinical aging populations.

## CONFLICT OF INTERESTS

The authors declare that the research was conducted in the absence of any commercial or financial relationships that could be construed as a potential conflict of interest.

## AUTHOR CONTRIBUTIONS

Susan Mérillat, Franziskus Liem, and Lutz Jäncke contributed to the design, set‐up, maintenance, and support of the Longitudinal Healthy Aging Brain (LHAB) database. Brigitta Malagurski performed the data analysis and wrote the first draft of the manuscript. All authors discussed the results, contributed to manuscript revision, read, and approved the submitted version.

## Supporting information


**Appendix** **S1** Supporting Information.

## Data Availability

The data for this manuscript are not publicly available. Since data collection was started in 2011, when public data sharing and open science were not yet widely discussed, the used consent does not allow for the public sharing of the data. We are currently working on a solution for this matter. At the moment, data can only be accessed via collaborations with the URPP “Dynamics of Healthy Aging”.
