# Supplementary material for: Longitudinal functional brain network reconfiguration in healthy aging
Source: Hum Brain Mapp. 2020 Aug 28;41(17):4829–45. doi: 10.1002/hbm.25161 (PMC7643380; doi:10.1002/hbm.25161)
Supplement: Supplementary file 1 — Appendix S1 Supporting Information. [file HBM-41-4829-s001.docx]

**
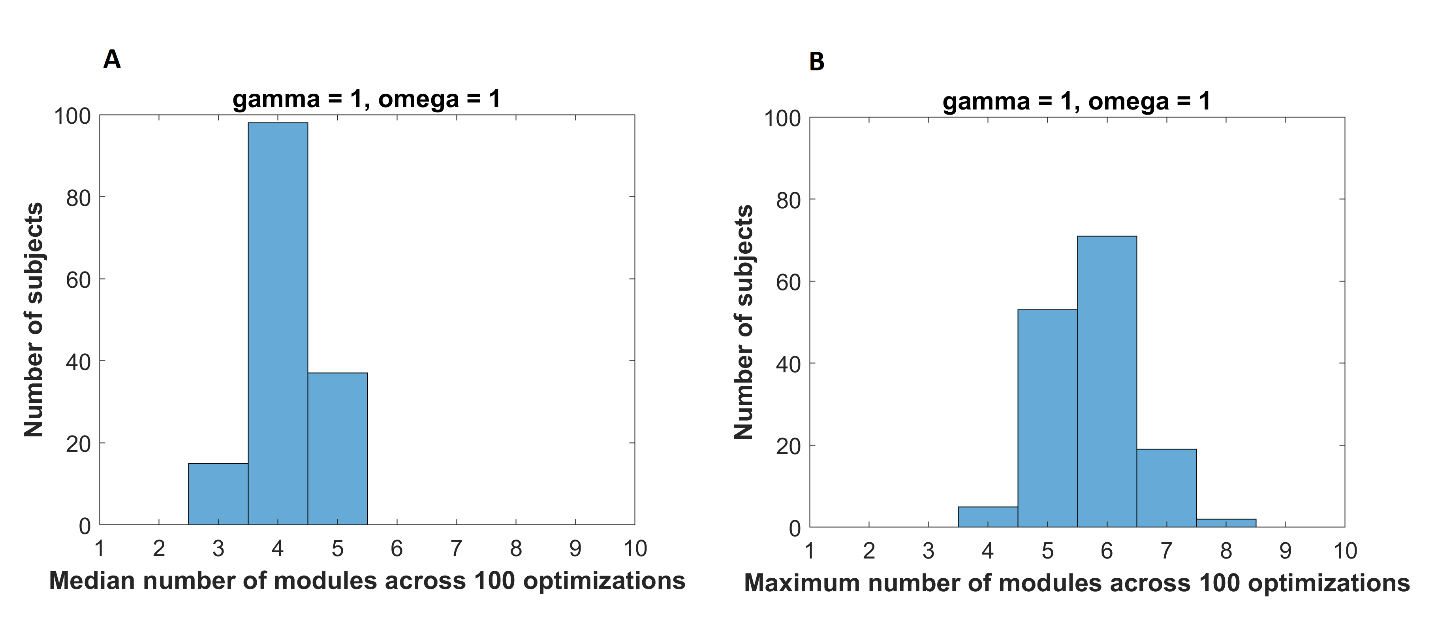
**

**Supplementary figure 1.** The histograms show median (A) and maximum (B) number of modules across 100 optimizations of the multilayer modularity algorithm.


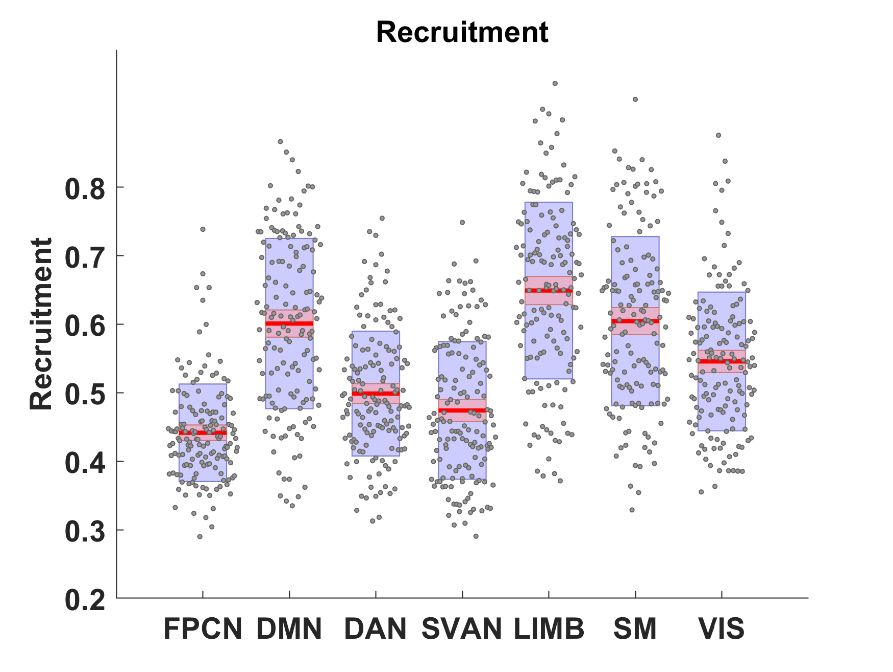


**Supplementary figure 2.** Visualization of summary statistics for network-specific recruitment across the 4-year interval. The center red lines represent the mean, and the light red bars and light blue bars represent 95% confidence interval and standard deviation, respectively. This figure was generated using notBoxPlot (<https://github.com/raacampbell/notBoxPlot>). FPCN – frontoparietal control; DMN – default mode; DAN – dorsal attention network; SVAN – salience ventral attention network; LIMB – limbic network; SM- somatomotor; VIS – visual networks. While all resting state networks are consistently recruited as network communities across the 4-year interval, we can see that networks vary in the strength of self-recruitment. Some networks, such as the SM, VIS, LIMB networks are consistently highly recruited, while other network such as the FPCN, DAN, SVAN are inconsistently recruited across the state time interval.

**
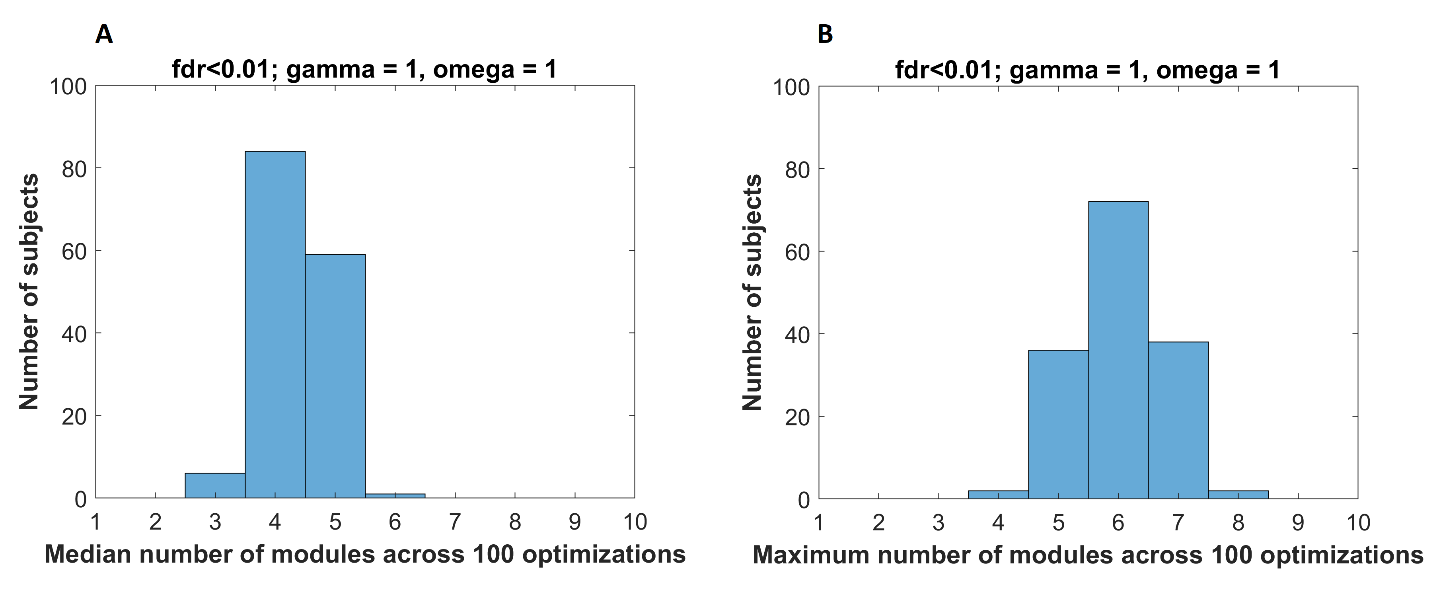
**

**Supplementary figure 3**. The histograms show median (A) and maximum (B) number of modules across 100 optimizations of the multilayer modularity algorithm using the thresholded matrices at FDR-adjusted p<0.01.


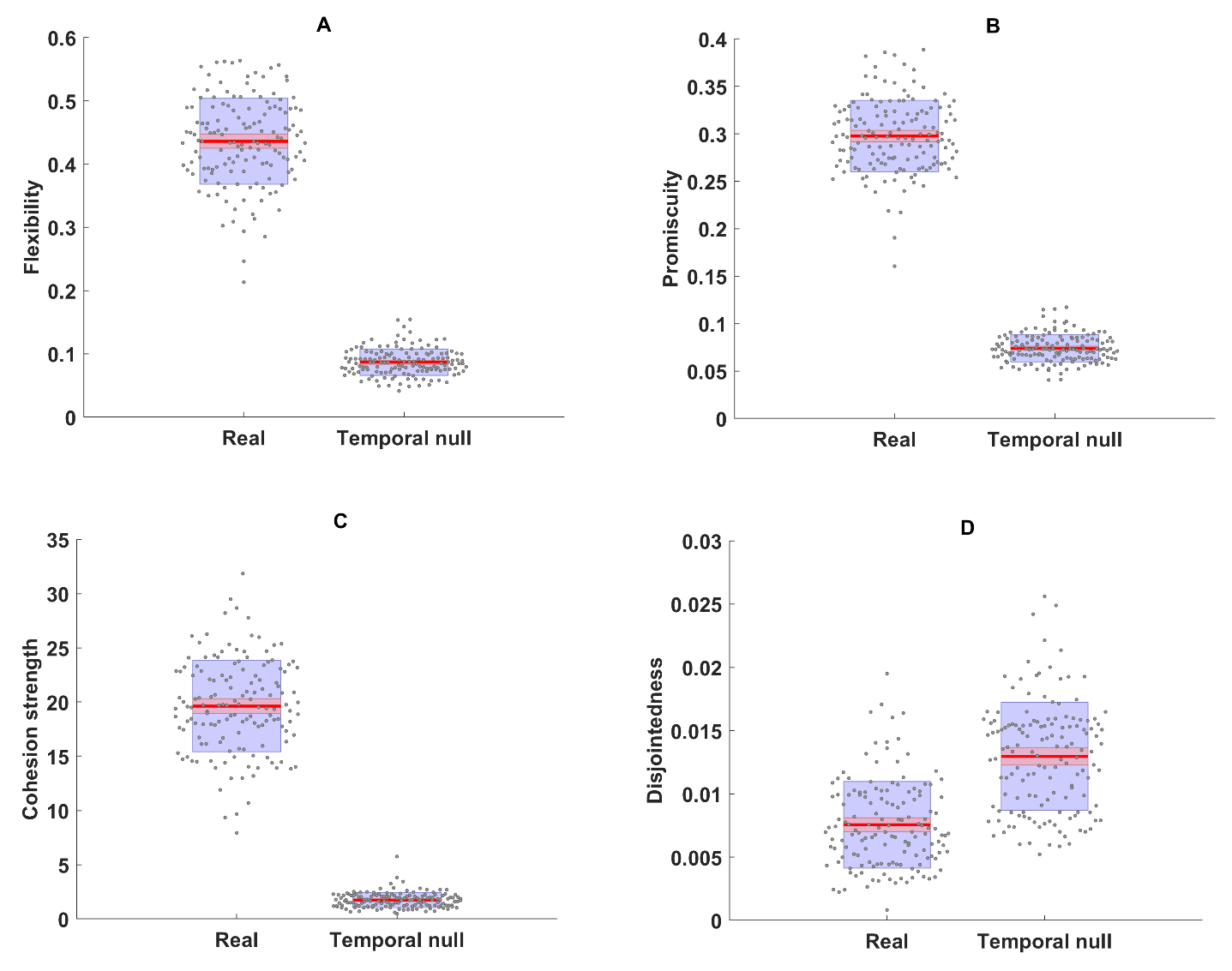


**Supplementary figure 4.** Comparison of global flexibility (A), promiscuity (B), cohesion strength (C) and disjointedness (D) of real networks (thresholded at FDR-adjusted p<0.01), to a temporal null model. The observed flexibility (t(298)=60.2, p<0.0001, d=6.97), promiscuity (t(298)=68.1, p<0.0001, d=7.87), and cohesion strength (t(298)=51.2, p<0.0001, d=5.94) were significantly higher than in the temporal null model. In contrast, disjointedness (t(298)=-12.2, p<0.0001, d=1.41) was significantly lower than in the temporal null model.


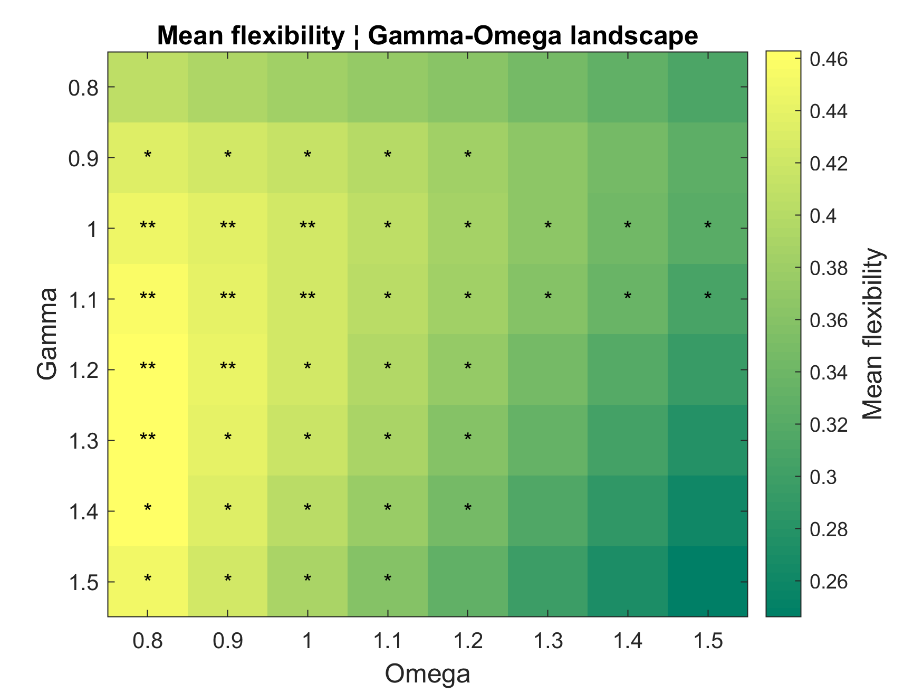


**Supplementary figure 5.** Multiple regression models with global flexibility with varying resolution parameters. We varied the structural (ɣ) and temporal (⍵) parameters over a range of 0.8-1.5 with increments of 0.1. For each pair of parameters, we performed multilayer community detection as described in the main text. We performed multiple regression analysis (as described in the main text) with calculated flexibility in order to assess the association between this measure and age at baseline. Here we show the significance level (p value) of the association between a given measures and age. The color scale presents the p value. * p<0.05, ** p<0.01.


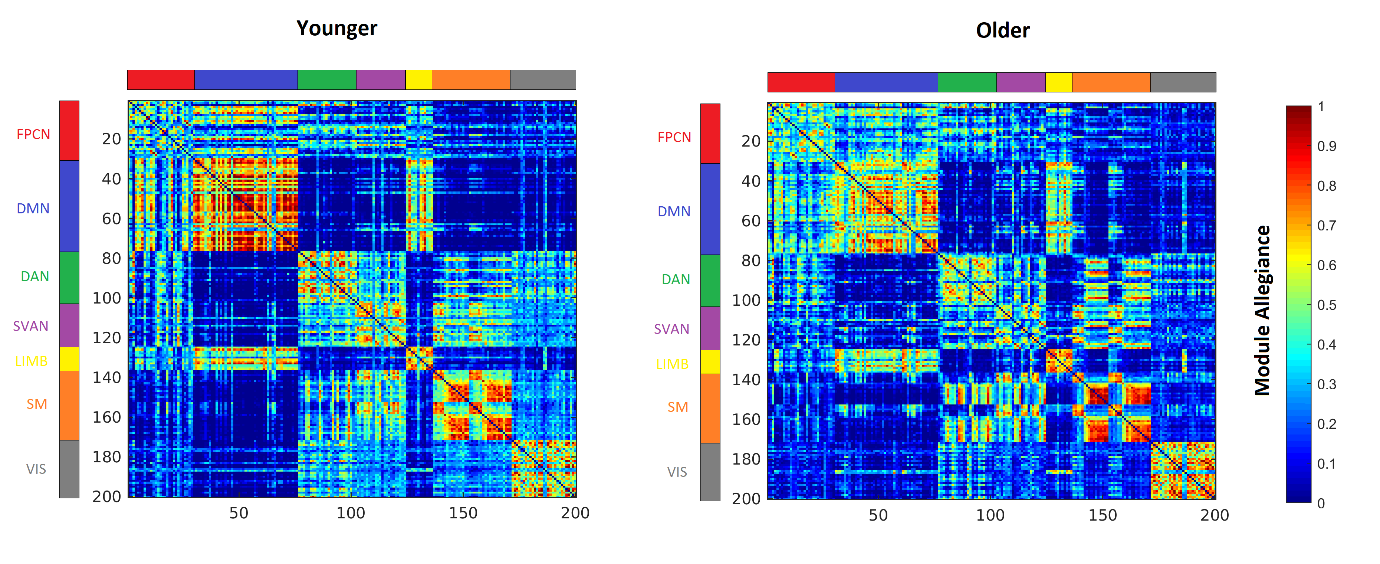


**Supplementary figure 6.** The module allegiance matrix was calculated for the youngest 10% (N = 15, M = 64.9y, range = 64 – 65.2y) and for the oldest 10% (N = 15, M = 78.7y, range = 76 – 82.6y) of the sample. It is noticeable that networks from the Schaefer atlas tend to be recruited together in the same communities (across the 4-year interval) more frequently in the younger versus the older subgroup. This is especially visible for the DMN network, which is compatible with our results showing lower self-recruitment of this network with older age.


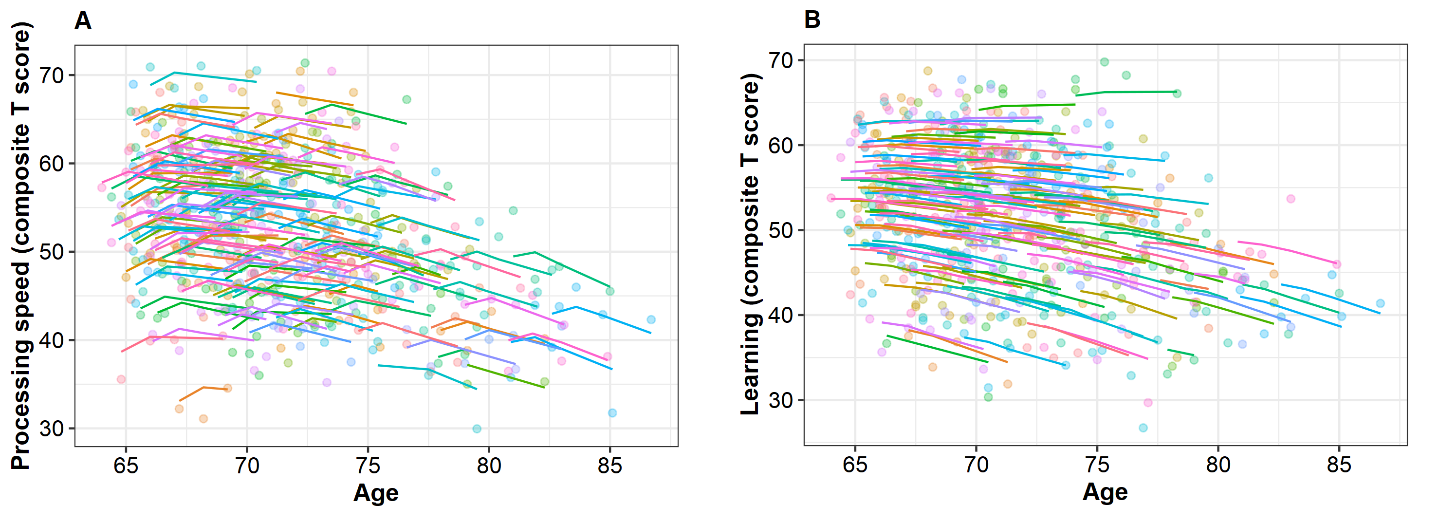


**Supplementary figure 7.** Spaghetti plots of model-fitted longitudinal changes in processing speed (A) and learning/memory encoding (B) for each individual.

**Supplementary table 1.** Participant characteristics of the full sample at baseline and at the sample with no missing data at 4-year follow-up.

| Variable | Baseline sample  ( n = 232) | | | Sample with no missing data  (n = 150) | | | Total selectivity |
| --- | --- | --- | --- | --- | --- | --- | --- |
|  | n | M | SD | n | M | SD |  |
| Age (years) | 232 | 70.84 | 5.08 | 150 | 69.84 | 4.18 | -0.20 |
| Gender (% f) | 232 | 49.14 | - | 150 | 47.33 | - | - |
| Education (1-3) | 226 | 2.23 | 0.86 | 149 | 2.31 | 0.82 | 0.09 |
| Processing speed at baseline | 224 | 50.13 | 8.35 | 148 | 51.72 | 8.25 | 0.19 |
| Learning at baseline | 227 | 50.09 | 8.52 | 147 | 51.78 | 7.77 | 0.20 |
| Mental health | 213 | 54.77 | 6.25 | 140 | 54.87 | 5.73 | 0.02 |
| Physical health | 213 | 50.79 | 7.42 | 140 | 51.45 | 6.38 | 0.09 |

*Note*. f = female. Education was measured on a scale from 1 to 3 (1 = high school with or without vocational education, 2 = higher education entrance qualification, business school or university of applied sciences, or 3 = university degree). Individual cognitive task scores were standardized to T scores (M=50, SD=10) with respect to baseline and averaged across subtests to calculate the domain-average composite scores. Mental and physical health scores were computed based on the SF12 questionnaire, which participants filled out at home (Ware, Kosinski, & Keller, 1996). Total selectivity was computed for the baseline sample as compared to the baseline values of the sample with no missing rs-fMRI data at 4-y follow-up (M_4-y_-M_base_/SD_base_).

**Supplementary Table 2. Association between network-specific flexibility and age at baseline in multiple regression models.** Models that did not show a significant association between network flexibility and age at baseline.

| *Network* | *Predictors* | *Estimates* | *SE* | *CI* | *p* | *Partial η^2^* |
| --- | --- | --- | --- | --- | --- | --- |
|  | (Intercept) | 0.4804 | 0.0268 | 0.4278 – 0.5330 | **<0.001** | **0.6883** |
| **DAN** | Gender | 0.0020 | 0.0147 | -0.0268 – 0.0308 | 0.893 | 0.0001 |
|  | Education | -0.0001 | 0.0092 | -0.0181 – 0.0178 | 0.989 | <0.0001 |
|  | Age | 0.0027 | 0.0017 | -0.0007 – 0.0060 | 0.125 | 0.0162 |
|  | FD | -0.1448 | 0.0683 | -0.2787 – -0.0109 | **0.036** | **0.0300** |
|  |  |  |  |  |  |  |
|  | (Intercept) | 0.4895 | 0.0247 | 0.4411 – 0.5378 | **<0.001** | **0.7310** |
| **SVAN** | Gender | -0.0084 | 0.0135 | -0.0348 – 0.0180 | 0.535 | 0.0027 |
|  | Education | 0.0049 | 0.0084 | -0.0116 – 0.0214 | 0.561 | 0.0023 |
|  | Age | 0.0016 | 0.0016 | -0.0015 – 0.0047 | 0.304 | 0.0073 |
|  | FD | -0.0081 | 0.0628 | -0.1311 – 0.1149 | 0.898 | 0.0001 |
|  |  |  |  |  |  |  |
|  | (Intercept) | 0.4162 | 0.0416 | 0.3348 – 0.4977 | **<0.001** | **0.4090** |
| **LIMB** | Gender | -0.0215 | 0.0227 | -0.0660 – 0.0231 | 0.346 | 0.0061 |
|  | Education | 0.0071 | 0.0142 | -0.0207 – 0.0349 | 0.619 | 0.0017 |
|  | Age | 0.0003 | 0.0027 | -0.0049 – 0.0055 | 0.906 | 0.0001 |
|  | FD | -0.0003 | 0.1058 | -0.2076 – 0.2070 | 0.998 | <0.0001 |
|  |  |  |  |  |  |  |
|  | (Intercept) | 0.4580 | 0.0310 | 0.3973 – 0.5188 | **<0.001** | **0.6009** |
| **VIS** | Gender | -0.0008 | 0.0170 | -0.0340 – 0.0325 | 0.964 | <0.0001 |
|  | Education | 0.0101 | 0.0106 | -0.0106 – 0.0309 | 0.340 | 0.0063 |
|  | Age | -0.0002 | 0.0020 | -0.0041 – 0.0037 | 0.926 | 0.0001 |
|  | FD | -0.0380 | 0.0789 | -0.1926 – 0.1167 | 0.631 | 0.0016 |

*Note.* FD – average framewise displacement across 4 time points. DAN – dorsal attention network, SVAN – salience ventral attention network, LIMB – limbic network, VIS – visual network.

**Supplementary Table 3. Association between network-specific promiscuity and recruitment and age at baseline in multiple regression models.** Statistically significant effects (p<0.05) appear in bold.

| *Network* | *Predictors* | *Estimates* | *SE* | *CI* | *p* | *Partial η^2^* |
| --- | --- | --- | --- | --- | --- | --- |
|  | (Intercept) | 0.2545 | 0.0203 | 0.2144 – 0.2947 | **<0.001** | **0.5198** |
| **DMN Promiscuity** | Gender | -0.0233 | 0.0111 | -0.0453 – -0.0014 | **0.038** | **0.0295** |
|  | Education | 0.0074 | 0.0069 | -0.0063 – 0.0211 | 0.289 | 0.0077 |
|  | Age | 0.0041 | 0.0013 | 0.0015 – 0.0066 | **0.002^*^** | **0.0627** |
|  | FD | -0.0393 | 0.0517 | -0.1415 – 0.0629 | 0.448 | 0.0040 |
|  | (Intercept) | 0.5520 | 0.0356 | 0.4816 – 0.6224 | **<0.001** | **0.6234** |
| **DMN Recruitment** | Gender | 0.0673 | 0.0195 | 0.0287 – 0.1058 | **0.001^*^** | **0.0759** |
|  | Education | -0.0071 | 0.0122 | -0.0312 – 0.0169 | 0.558 | 0.0024 |
|  | Age | -0.0063 | 0.0023 | -0.0108 – -0.0018 | **0.006^*^** | **0.0501** |
|  | FD | 0.1448 | 0.0907 | -0.0345 – 0.3240 | 0.113 | 0.0173 |
|  | (Intercept) | 0.6414 | 0.0361 | 0.5700 – 0.7128 | **<0.001** | 0.6848 |
| **SM Recruitment** | Gender | -0.0375 | 0.0198 | -0.0765 – 0.0016 | 0.060 | 0.0242 |
|  | Education | -0.0163 | 0.0123 | -0.0407 – 0.0081 | 0.189 | 0.0119 |
|  | Age | -0.0085 | 0.0023 | -0.0131 – -0.0039 | **<0.001^*^** | **0.0851** |
|  | FD | 0.0807 | 0.0920 | -0.1011 – 0.2624 | 0.382 | 0.0053 |

*Note.* FD – average framewise displacement across 4 time points. * survives multiple comparison correction (p<0.05 corrected for 9 tests). DMN – default mode network, SM – somatomotor network.

**Brain-cognition change-change association**

Pearson’s correlation analysis was calculated to assess the association between global and network-specific flexibility and change in cognitive performance.

We did not find a significant relationship between change in processing speed and any of the network-specific flexibility metrics - the DMN (r(148) = 0.02, 95% CI [-0.14, 0.18], p = 0.79), FPCN (r(148) = -0.06, 95% CI [-0.22, 0.10], p = 0.44), or SM network flexibility (r(148) = -0.03, 95% CI [-0.19, 0.13], p = 0.74). Also, there were no significant associations between longitudinal change in learning/memory encoding and individual network flexibility: DMN (r(146) = -0.05, 95% CI [-0.21, 0.11], p = 0.52), FPCN (r(146) = -0.05, 95% CI [-0.21, 0.12], p = 0.58) or SM network flexibility (r(146) = -0.03, 95% CI [-0.19, 0.13], p = 0.74). Running the partial correlation analysis in the aim of controlling the effects of age at baseline did not change these results.

The brain-cognition association was tested for all other brain networks as well (which did not have a significant association with age at baseline), and there were no significant relationships found between any of the remaining networks (i.e. DAN, SVAN, LIMB, VIS) and processing speed or learning/memory encoding.
